# Histological criteria for selecting patients who need clonality test for non-gastric MALT lymphoma diagnosis

**DOI:** 10.1186/s13000-024-01471-8

**Published:** 2024-03-08

**Authors:** Dajeong Park, Junhun Cho

**Affiliations:** Department of Pathology, Samsung Medical Center, Sungkyunkwan University School of Medicine, #81, Irwon-ro, Gangnam-Gu, Seoul, 06351 Korea

**Keywords:** Extranodal marginal zone lymphoma of MALT, MALT lymphoma, Marginal zone expansion, Digital pathology

## Abstract

The histological diagnosis of extranodal marginal zone lymphoma of the mucosa-associated lymphoid tissue (MALT lymphoma) is difficult for pathologists. Recently, digital pathology systems have been widely used to provide tools that can objectively measure lesions on slides. In this study, we measured the extent of marginal zone expansion in suspected MALT lymphoma cases and compared the results with those of a molecular clonality test. In total, 115 patients who underwent an IGH gene rearrangement test for suspected MALT lymphoma were included in this study. All cases were histologically classified into three patterns; “small lymphoid aggregates with no germinal center (Pattern 1),” “lymphoid follicles with germinal center (Pattern 2)” and " fused marginal zone or diffuse small lymphocytic proliferation (Pattern 3).” The proportions of monoclonality in Pattern 1, 2, and 3 were 25.0%, 55.0%, and 97.9%, respectively. The ratios of marginal zone thickness to germinal center diameter and entire lymphoid follicle area to germinal center area were measured in Pattern 2 cases using a digital pathology system. Combining the width cutoff of 1.5 and the areal cutoff of 3.5, the sensitivity, specificity, positive predictive value, and negative predictive value for MALT lymphoma were 96.97%, 70.37%, 80.00%, and 95.00%, respectively. In conclusion, through objective measurement of the marginal zone, suspected cases of MALT lymphoma requiring a molecular clonality test can be effectively selected.

**Supplementary Information:**

The online version contains supplementary material available at 10.1186/s13000-024-01471-8.

## Introduction

Extranodal marginal zone lymphoma of mucosa-associated lymphoid tissue (MALT lymphoma) is a low-grade extranodal B-cell lymphoma arising from memory B lymphocytes in the marginal zone of secondary lymphoid follicles [1, 2]. MALT lymphoma constitutes 5–17% of all non-Hodgkin lymphomas [3], and represents the most common low-grade B-cell lymphoma. Its incidence is slightly higher in Asians, accounting for 16.7% of all non-Hodgkin lymphomas in Korea, probably because of the high prevalence of Helicobacter pylori infections [4]. The most commonly affected sites of MALT lymphoma are stomach, salivary glands, skin, orbits, conjunctiva, lung, thyroid, breast, and other intestinal sites [4–6]. 

Although MALT lymphoma is one of the most commonly encountered lymphoproliferative disorders in daily practice, its diagnosis often poses a challenge to pathologists who are inexperienced in examining lymphoproliferative lesions. This is because of difficulties in judging whether the expansion of the marginal zones is neoplastic or reactive, as MALT lymphoma mimics reactive mucosa-associated lymphoid tissue (MALT). The presence of lymphoepithelial lesions (LELs), one of the diagnostic criteria of MALT lymphoma defined as ≥ 3 marginal zone cells with destruction of epithelial cells [1, 7], is often difficult to determine in small biopsies, and is not always specific to MALT lymphoma as it can represent variety of other benign inflammatory or autoimmune conditions such as IgG4-related disease, Sjögren syndrome, and nonspecific chronic inflammation [8–11]. There are no specific immunohistochemical markers for MALT lymphoma, and B-cell lineage markers can provide little help, except that it is a B-cell proliferative lesion. Therefore, in many cases, verification of the presence of B-cell clonality using immunoglobulin heavy chain (IGH) gene rearrangement tests is required to diagnose MALT lymphoma. However, performing an IGH gene arrangement test in every case of suspected MALT lymphoma is time- and cost-consuming. Therefore, there is a need to establish a definitive criteria for molecular clonality testing.

In this study, we attempted to suggest histological criteria for performing B-cell clonality test in cases exhibiting morphological features suggestive of non-gastric MALT lymphoma by retrospectively reviewing the histological slides of cases in which IGH gene rearrangement tests were performed.

## Materials and methods

### Patient selection

A total of 115 patients with suspected MALT lymphoma who underwent the IGH gene rearrangement test (EuroClonality/BIOMED-2) between November 2014 and June 2022 at Samsung Medical Center, Seoul, Korea, were included in the study. Cases in which the test results were insufficient for diagnosis due to poor DNA quality were also excluded from the study. There were 56 males and 59 females (male-to-female ratio: 1:1.05). The median age was 55 years (range, 11–83 years). There were 69 eyelid, 16 skin, 16 lung, and 14 intestine tissues. Stomach cases were not included in this study because most MALT lymphomas arising from the stomach are diagnosed without clonality testing at our institute. The IGH tests were interpreted by an experienced hematopathologist (J.C.) according to the EuroClonality/BIOMED-2 guidelines [12]. Duplicate test was performed in all cases. As a result of the tests, monoclonality was detected in 81 cases (70.4%) and 34 cases were polyclonal. Of the 81 cases, seven cases showed a low intensity peak, but the peak of the same base pair size was reproduced in the duplication test and were classified as monoclonal cases. Clinicopathological characteristics are summarized in Table 1. All methods were carried out in accordance with Helsinki declaration, and all protocols of this study were approved by the Institutional Review Board of Samsung Medical Center (IRB file number: SMC 2021-01-093).

**Table 1:** Clinical characteristics of 115 patients

		Polyclonal (%) (*N* = 34)	Monoclonal (%) (*N* = 81)	Total
Sex	Male	14 (25.0)	42 (75.0)	56
	Female	20 (33.9)	39 (66.1)	59
Age	< 60	21 (30.0)	49 (70.0)	70
	≥ 60	13 (28.9)	32 (71.1)	45
Organ	Eyelid	16 (23.2)	53 (76.8)	69
	Lung	5 (31.3)	11 (68.8)	16
	Skin	7 (43.8)	9 (56.3)	16
	Intestine	6 (42.9)	8 (57.1)	14

### Immunohistochemistry

To accurately evaluate the structures of the lymphoid follicles, including the germinal center and marginal zone, CD3 (polyclonal, Dako, CA, USA), CD20 (clone L26, Novocastra, Leica Biosystems, Germany), and Ki-67 (clone MIB1, Dako, CA, USA) immunohistochemical staining was performed on whole sections of formalin-fixed paraffin-embedded blocks in all cases. Details of immunohistochemical staining are provided in Supplementary Table 1.

### Histological review

All hematoxylin and eosin (H&E) and immunohistochemical slides were scanned using a Pannoramic 1000 Slide Scanner (3DHISTECH) and reviewed using a digital slide viewer (INFINITT Digital Pathology Solution). One pathology resident (D.P.) conducted the initial examination of every slide, all of which were subsequently reviewed by an experienced hematopathologist (J.C.).

For each case, the well-formed lymphoid follicle with the largest marginal zone expansion on the H&E slide was selected as the representative follicle for measurement. If lymphoid proliferation without germinal center formation was observed, the cases were excluded from measurement. Additionally, cases in which the expansion of marginal zone B cells was so marked that the adjacent marginal zones were diffusely fused together, rendering it impossible to distinguish each individual lymphoid follicle, were also excluded.

For each selected lymphoid follicle, the area of the entire lymphoid follicle and its germinal center were measured using the automatic area measurement function of a digital slide viewer. The ratio of the area of the entire lymphoid follicle to that of the germinal center was calculated. Next, the width of the germinal center and thickness of the marginal zone were measured using the ruler function of the digital slide viewer. As the morphology and shape of lymphoid follicles are not always symmetrical or regular, we selected a reference point starting from the corner of the thickest part of the marginal zone and then drew a straight line across the lymphoid follicle, ending at the very center of the germinal center. On such a straight line, the width occupied by the marginal zone was considered the thickness of the marginal zone, and the width occupied by the germinal center was considered the radius of the germinal center. The ratio of the marginal zone thickness to the germinal center radius was also calculated.

## Results

### Morphological patterns and IGH gene rearrangement test

**Fig. 1:**
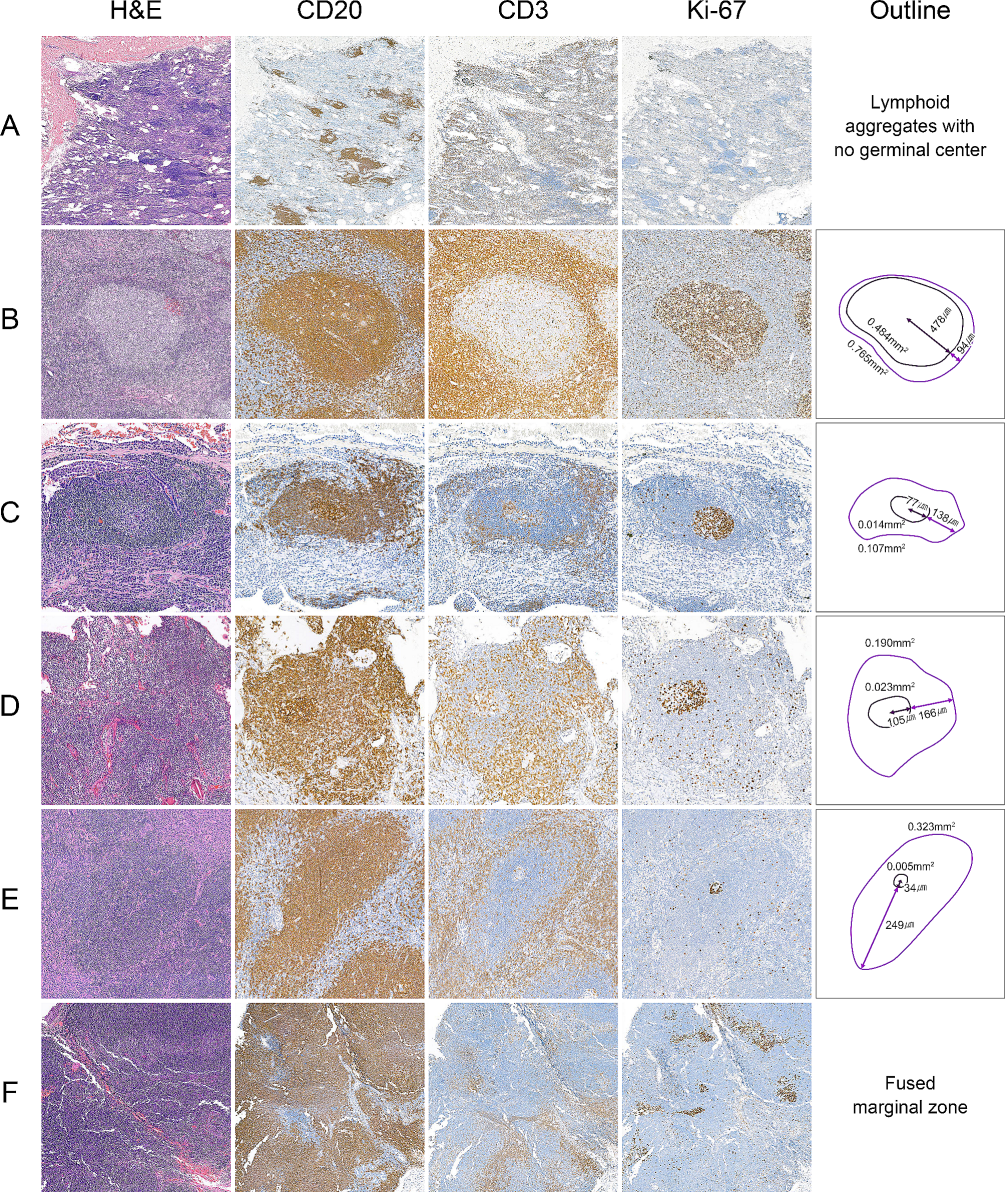
Figures of hematoxylin and eosin stains, immunohistochemistries (CD3, CD20, and Ki-67) and schematic images showing the stepwise expansion of marginal zone. A) Pattern 1: small lymphoid aggregates with no germinal center formation; B-E) Pattern 2: lymphoid follicles with germinal center; F) Pattern 3: fused marginal zone or diffuse small lymphocytic proliferation. Expansion of marginal zone was measured only in the Pattern 2 cases (B-E). H&E, hematoxylin and eosin

Three morphological patterns were observed upon histological evaluation. Pattern 1, “small lymphoid aggregates with no germinal center”, are represented by cases showing multifocal small lymphoid aggregates but without the presence of a germinal center revealed by Ki-67 staining (*n* = 8) (Fig. 1A). Pattern 2 is defined as “lymphoid follicles with a germinal center” in their center (*n* = 60) (Fig. 1B-E). Pattern 3, “fused marginal zone or diffuse small lymphocytic proliferation”, is a lesion with diffuse lymphoid proliferation in which immunohistochemical staining reveals merged marginal zones (*n* = 47) (Fig. 1F). As a result of the IGH gene rearrangement tests, monoclonality was 25.0% (2/8) in Pattern 1, 55.0% (33/60) in Pattern 2, and 97.9% (46/47) in Pattern 3. Measuring the areas or widths of the marginal zone and germinal center was only possible in Pattern 2 cases.

### Area (ratio of the entire lymphoid follicle to the germinal center)

The area of the germinal centers of 60 cases was 0.0793mm^2^ in average (range, 0.0047–0.4835). The average area of the lymphoid follicles, including the area of the germinal centers, was 0.3222 mm^2^ (range, 0.0264–1.0683). The mean value of the areal ratio of the lymphoid follicles to the germinal centers was 7.06, with the median value of 4.54 (range, 1.46–69.39).

**Fig. 2:**
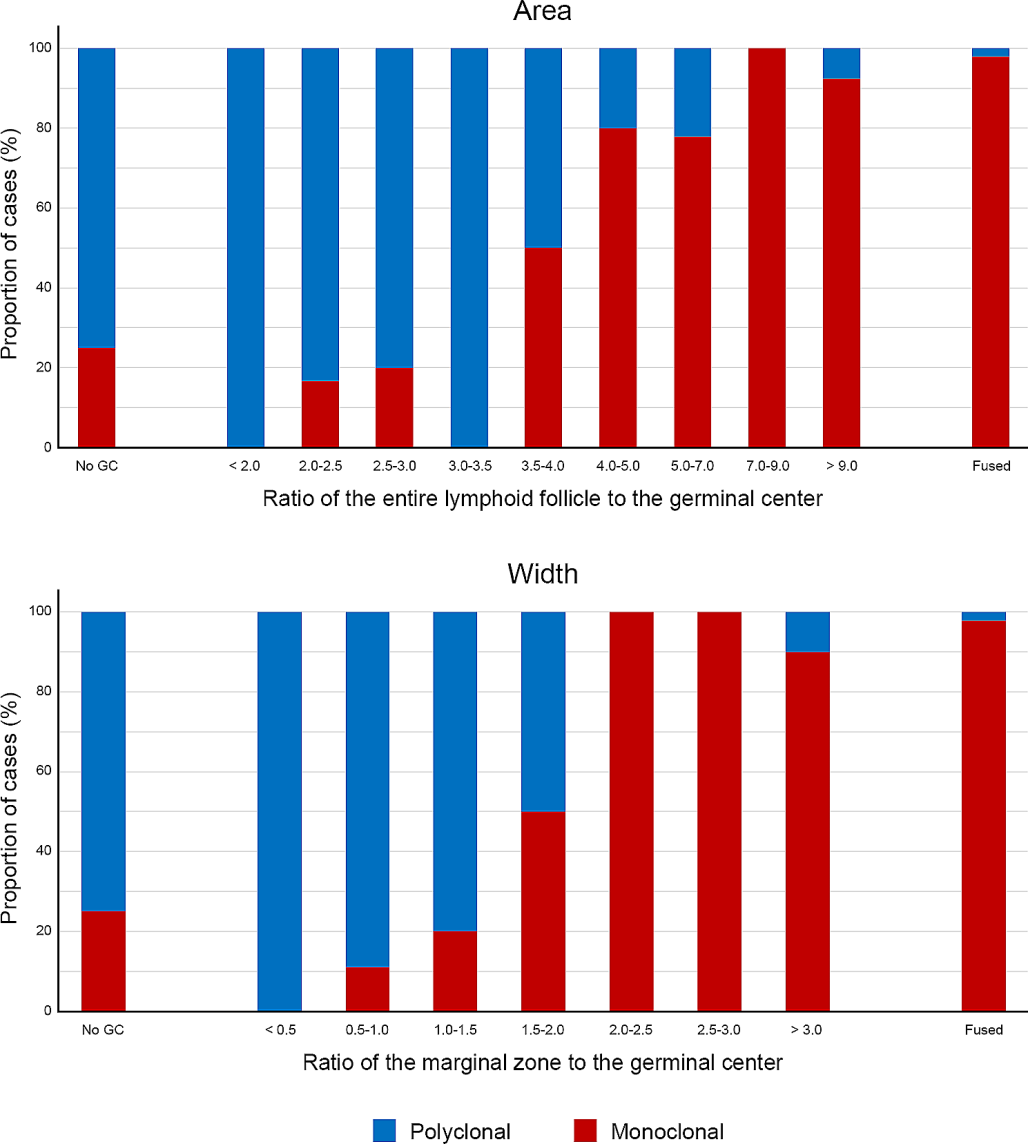
Bar graphs showing the proportion of cases with monoclonality by area (ratio of the entire lymphoid follicle to the germinal center) and width (ratio of the marginal zone to the germinal center)

Figure 2A demonstrates the distribution of the areal ratio of the germinal center to the lymphoid follicle at intervals of 0.5, and the proportion of monoclonal and polyclonal cases in each interval section. The ratio less than 2.0 is entirely composed of monoclonal cases. There are more polyclonal cases than monoclonal in the 2.0-3.5 interval. In the 3.5-4.0 interval, the number of monoclonal and polyclonal cases is the same. The number of monoclonal cases started to exceed the number of polyclonal cases above a ratio of 4.0.

### Width (ratio of the marginal zone to the germinal center)

The measured radius of the germinal centers was 152.2 μm in average (range, 34–412 μm). The thickness of the marginal zones was 251.6 μm in average (range, 53–555 μm). The mean value of the ratio of the widths of the marginal zone to the germinal center was 2.06, with median value of 1.75 (range, 0.29–7.32).

Figure 2B shows the distribution of the ratio of the widths of the marginal zone of the germinal center at intervals of 0.5, with the proportion of monoclonal and polyclonal cases. All cases with a ratio below 0.5, were diagnosed as polyclonal by clonality test. In the 0.5–1.5 interval, the number of polyclonal cases exceeded the number of monoclonal cases. The number was the same in the 1.5-2.0 interval. The ratio above 2.0. almost entirely consisted of monoclonal cases.

### Suggestion of histological criteria

Based on the above findings, we sought to find histological criteria for selecting cases requiring clonality test among cases of suspected MALT lymphoma. For the areal ratio of the entire lymphoid follicle to the germinal center, a 3.5-cutoff value has a sensitivity of 93.94% and specificity of 74.07% in diagnosing MALT lymphoma. The positive predictive value (PPV) and negative predictive value (NPV) are 81.58% and 90.91%, respectively. As for the ratio of the widths of the marginal zone to the germinal center, a 1.5-cutoff has a sensitivity, specificity, PPV, and NPV of 90.91%, 81.48%, 85.71%, and 88.00%, respectively, in the diagnosis of MALT lymphoma. Combining the above two criteria, when conducting the IGH gene rearrangement test in cases that meet the areal cutoff of 3.5 or width cutoff of 1.5, the sensitivity, specificity, PPV, and NPV for MALT lymphoma are 96.97%, 70.37%, 80.00%, and 95.00%, respectively.

## Discussion

The diagnosis of MALT lymphoma has always been difficult for pathologists because of its morphological similarity to reactive mucosa-associated lymphoid tissue (MALT), minimal cytological atypia, and lack of specific markers. In stomach, Wotherspoon grade [13] has been proposed and used to predict the possibility of MALT lymphoma. However, histological diagnostic criteria for MALT lymphomas in other extranodal organs remain unclear. Therefore, the molecular clonality test is essential for the diagnosis of MALT lymphoma; however, the criteria for performing the molecular clonal test are not clear. This study presented histological criteria to select suspected MALT lymphoma cases requiring molecular clonality tests through precise histology analysis using a digital slide viewer and IGH gene rearrangement test results, excluding stomach cases.

MALT lymphoma is caused by the oncogenic cooperation between dysregulated immune responses and somatic genetic changes resulting from a chronic inflammatory process [14, 15]. Various translocations are observed depending on the organ in which they occur [16, 17], most of which are associated with the activation of the NF-κB oncogenic pathway [18]. As a result, MALT lymphoma occurs through monoclonal proliferation of marginal zone B cells. Therefore, determining whether the marginal zone is expanded and to what extent, which is the most important part of the pathological diagnosis of MALT lymphoma. However, it is difficult to determine whether the marginal zone expansion has progressed beyond reactive changes in neoplasms during pathological diagnosis. To date, numerous studies have described the histological findings of MALT lymphoma [19–23]. MALT lymphomas that arise in various organs share similar histological features. Neoplastic cells are small-to medium-sized lymphoid cells that infiltrate reactive secondary lymphoid follicles and spread outwards to form a diffuse interfollicular sheet or vague nodular pattern. Germinal centers can be replaced by lymphoma cells. Depending on the site, lymphoepithelial lesions can be observed. Neoplastic cells may have a monocytoid appearance, with an abundant pale cytoplasm and distinct cell borders. Variable numbers of plasma cells are frequently present.

In this study, the proportion of monoclonality was low in cases with small lymphoid aggregates without GC (25.0%), and very high in cases with fused marginal zones of different follicles or a diffuse pattern (97.9%). In lymphoid follicles, where the germinal center was observed and the marginal zones were not fused with each other, we precisely measured the ratio of the area of the entire follicle to the germinal center and the ratio of the thickness of the marginal zone to the diameter of the germinal center using a digital slide viewer. Through this, a diameter cut-off of 1.5 and an area cut-off of 3.5 were presented as criteria for the molecular clonality test. The sensitivity for monoclonality reached 96.97% when the two criteria were combined, demonstrating that this criterion is a valuable screening tool.

The recent widespread use of digital pathology has provided a tool for pathologists to analyze pathology images as digital data. Pathologists have previously evaluated the degree of marginal zone expansion for the diagnosis of MALT lymphoma using a microscope; however, the criteria are subjective and depend heavily on the pathologist’s personal experience. Although experienced hematopathologists are able to select MALT lymphoma-suspicious patients requiring a molecular clonality test, differential diagnosis has been a major challenge for general pathologists. Our study objectively suggests histological criteria for screening for neoplastic marginal zone expansion. In addition, by utilizing H&E staining and a small number of essential immunostains (CD3, CD20, and Ki-67), we present a method that allows general pathologists to recognize and accurately measure the expansion of the marginal zone. Even in an institute where a digital pathology system cannot be utilized, although it is difficult to measure the area, it is expected that it will be possible to intuitively compare the diameter of the germinal center and thickness of the marginal zone using a microscope, which will help select patients who need a molecular clonality test. Of course, it should not be forgotten that excluding the possibility of other lymphomas, such as follicular lymphoma, mantle cell lymphoma, and chronic lymphocytic leukemia/small lymphocytic lymphoma, is important in diagnosing MALT lymphoma especially in cases with a diffuse pattern.

This study has several limitations. First, EuroClonality/BIOMED-2 recommended performing light chain tests (IGK and IGL) together with IGH test for best sensitivity [12], but light chain tests were not performed in this study. Second, the possibility of plasmacytic differentiation was not considered in the interpretation of the immunostaining. Some MALT lymphomas show plasmacytic differentiation [24] and the extent of the lesion may be underestimated by CD20 staining. In such cases, it is difficult to apply the criteria proposed in our study and it is necessary to confirm the restriction of plasma cells using kappa and lambda light chain staining.

In institutions where molecular clonality tests such as the IGH gene rearrangement test are not set up, the authors suggest a diagnosis of “Lymphoid hyperplasia with moderate marginal zone expansion” and an additional comment “Based on histology, the possibility of MALT lymphoma is raised. Additional molecular clonality tests may help in diagnosis.” Through this, clinicians will be able to closely follow-up patients or transfer them to other institutions.

In conclusion, in a biopsy of a patient with marginal zone expansion, the possibility of lymphoma was high when the marginal zones of different follicles were fused together or when small lymphocytes showed a diffuse pattern. In cases where distant lymphoid follicles are observed, if the thickness of the marginal zone compared to the diameter of the germinal center is more than 1.5 times, and the area of the entire lymphoid follicle is more than 3.5 times the area of the germinal center, the possibility of MALT lymphoma is high. Therefore, a clonality test is necessary in these cases. We hope that these results will be validated by further studies and that they will be widely used as histological criteria for diagnosing MALT lymphoma.

### Electronic supplementary material

Below is the link to the electronic supplementary material.

Supplementary Material 1

## Data Availability

No datasets were generated or analysed during the current study.
