# Supplementary material for: Histological criteria for selecting patients who need clonality test for non-gastric MALT lymphoma diagnosis
Source: Diagn Pathol. 2024 Mar 8;19:49. doi: 10.1186/s13000-024-01471-8 (PMC10921771; doi:10.1186/s13000-024-01471-8)
Supplement: Supplementary file 1 — Supplementary Material 1 [file 13000_2024_1471_MOESM1_ESM.docx]

**Supplementary Table 1.** The list of antibodies used for immunohistochemistry in this study

|  | **Antibodies** | **Cat. No.** | **Clone** | **Company** | **Dilution** | **Antigen retrieval** | **Endogenous peroxidase blocking** | **Incubation time with primary antibody** | **Secondary and chromogen** | **Machine** |
| --- | --- | --- | --- | --- | --- | --- | --- | --- | --- | --- |
| 1 | CD3 | A0452 | Polyclonal | DAKO | 1:200 | 20 min with ER1 Buffer (pH 6.0) in ROOM T. Bond-III | 5M | 15min with Bond-III autoimmunostainer (Leica Biosystem, Melbourne, Australia) | 8 min Bond-III autoimmunostainer (Leica Biosystem, Melbourne, Australia) using Bond™ Polymer refine detection, DS9800 (Vision Biosystems, Melbourne, Australia) | BOND III |
| 2 | CD20 | NCL-L-CD20-26 | L26 | Novocastra | 1:400 | 20 min with ER1 Buffer (pH 6.0) in ROOM T. Bond-III | 5M | 15min with Bond-III autoimmunostainer (Leica Biosystem, Melbourne, Australia) | 8 min Bond-III autoimmunostainer (Leica Biosystem, Melbourne, Australia) using Bond™ Polymer refine detection, DS9800 (Vision Biosystems, Melbourne, Australia) | BOND III |
| 3 | Ki-67 | M7240 | MIB1 | DAKO | 1:200 | 20 min with ER1 Buffer (pH 6.0) in ROOM T. Bond-III | 5M | 15min with Bond-III autoimmunostainer (Leica Biosystem, Melbourne, Australia) | 8 min Bond-III autoimmunostainer (Leica Biosystem, Melbourne, Australia) using Bond™ Polymer refine detection, DS9800 (Vision Biosystems, Melbourne, Australia) | BOND III |
